# Quad-PRE: A Hybrid Method to Predict Protein Quaternary Structure Attributes

**DOI:** 10.1155/2014/715494

**Published:** 2014-05-18

**Authors:** Yajun Sheng, Xingye Qiu, Chen Zhang, Jun Xu, Yanping Zhang, Wei Zheng, Ke Chen

**Affiliations:** ^1^School of Mathematical Sciences and LPMC, Nankai University, Tianjin 300071, China; ^2^School of Computer Science and Software Engineering, Tianjin Polytechnic University, No. 399 Binshui Road, Tianjin 300387, China

## Abstract

The protein quaternary structure is very important to the biological process. Predicting their attributes is an essential task in computational biology for the advancement of the proteomics. However, the existing methods did not consider sufficient properties of amino acid. To end this, we proposed a hybrid method Quad-PRE to predict protein quaternary structure attributes using the properties of amino acid, predicted secondary structure, predicted relative solvent accessibility, and position-specific scoring matrix profiles and motifs. Empirical evaluation on independent dataset shows that Quad-PRE achieved higher overall accuracy 81.7%, especially higher accuracy 92.8%, 93.3%, and 90.6% on discrimination for trimer, hexamer, and octamer, respectively. Our model also reveals that six features sets are all important to the prediction, and a hybrid method is an optimal strategy by now. The results indicate that the proposed method can classify protein quaternary structure attributes effectively.

## 1. Introduction

As is well known, the prediction of protein quaternary structure attributes (such as monomer, dimmer, trimer, tetramer, pentamer, hexamer, heptamer, and octamer) plays an important role in the structure bioinformatics. It can confirm how many subunits form the protein. It is the real requirement for the Anfinsen's dogma [1]. A variety of experimental techniques can determine protein quaternary structure. However, most methods are time-consuming and expensive. Moreover, the oligomers may be homooligomers or heterooligomers; the former consist of identical polypeptide chains, whereas the latter are nonidentical. Many computational methods are proposed.

As far as we know, the earliest work to study the quaternary structure type was in 2001 [2]. In this paper, Garian proposed a method named Quaternary Structure Explorer (QSE), which just judges whether or not a given protein is a homodimer. In 2003, Zhang et al. [3] first introduced support vector machine (SVM) to discriminate the differences of the primary sequences of both homodimer and nonhomodimer. Chou and Cai [4] solved the 2-state problem by using the pseudo amino acid composition. In 2006, Shi el al. [5] classified homooligomers based on amino acid composition distribution (AACD) and showed that the 2DPCA was an effective approach to decrease the high dimension of feature vector. In 2007, Carugo [6] proposed a method which is able to predict the quaternary structural type of hetero oligomeric proteins. Levy [7] proposed the PiQSi to get the annotations of about 15,000 proteins in PDB, which can be used as the benchmark dataset to test the quality of a method to predict the quaternary structure type. In 2009, Xiao and Lin introduced the grey incidence degree measure [8] to predict the protein quaternary structure attributes. The method is implemented as a web-server called Quat-2L [9], which firstly identifies the protein as homooligomer or heterooligomer and secondly justifies how many subunits. In 2012, Sun et al. utilized discrete wavelet transform [10] based on Chou's PseAAC to identify the protein quaternary structure attribute. All these methods to predict the quaternary structure attributes are based on one set of features, and mostly for 2 states.

In this paper, we proposed a new method Quad-PRE to predict protein quaternary structures attributes among 6 states only based on the primary sequences, removing both pentamer and heptamer because of insufficient data. With 10 fold cross validation, our models achieved higher overall accuracy 81.7%, especially higher accuracy 92.8%, 93.3%, and 90.6% on discrimination for trimer, hexamer, and octamer, respectively. Our method could be an effective tool to predict the protein quaternary structure attributes.

## 2. Materials and Methods

### 2.1. Benchmark Dataset

The dataset is from the quaternary structure library PiQSi (http://www.PiQSi.org/) built by Levy [7]. Our original dataset was downloaded on December 12, 2011. Firstly, we download a whole annotated list including about 15,000 protein sequences and a nonredundant set including 1755 sequences (30% sequence id.) from the library and then remove sequences which are not in the nonredundant set from the whole annotated list. In order to use a set of “good” PDB files, we use the subset of those annotated as “NOT” or “PROBABLY NOT” being errors. In addition, the number of pentamer and heptamer is too little to analyze and we also removed them. Finally, we get a protein quaternary structure dataset with primary sequence as shown in Table 1.

### 2.2. Features

In this paper, we used three traditional methods and three tools (BLAST, GLAM2, and GIBBS) to select 632 features only based on unique primary sequences and denoted them as six terms: ART_1 feature, ART_2 feature, ART_3 feature, BLAST feature, GLAM2 feature, and GIBBS feature). The summary of the considered features is shown in Table 2 (See Tables S1–S3 in Supplementary Material available online at http://dx.doi.org/10.1155/2014/715494 for more detailed information).

Firstly, we use three traditional methods to get the three feature sets, that is, the ART_1 feature by [12], ART_2 feature by [13], and ART_3 feature by [11], respectively. The sources of data used to generate the features from the original sequence include the protein sequence, the position-specific scoring matrix (PSSM) generated by PSI-BLAST [14], the secondary structure predicted by PSI-Pred [15], the solvent accessible surface area (ASA) values predicted using Real-SPINE [16], and the relative solvent accessibility (RSA) defined as the ratio of ASA of a residue observed in its three-dimensional structure to that observed in an extended (Gly-X-Gly or Ala-X-Ala) tripeptide conformation [17].

Secondly, we generate other three features sets by BLAST, GLAM2, and GIBBS, respectively. The three methods can describe the inherent properties of sequences. Primarily, we divide equally the feature set into 10 portions randomly, making sure that every portion contains at least one element of each one of 6 states (monomer, dimmer, trimer, tetramer, hexamer, and octamer) so that we have 10 datasets

(1) $$\begin{array}{c} \left\{S_{i}|\left|S_{i}\right|=104,i=1,\ldots ,10\right\}. \end{array}$$

Every *S*_*i*_ contains 6 subsets

(2) $$\begin{array}{c} S_{i}=\left\{p\;|\;p\in s_{ic},c=1,2,3,4,6,8\right\}, \end{array}$$

where each subset *s*_*ic*_ contains sequences which has *c* subunits in *S*_*i*_. It is noted that the generated features depend on the original 10 fixed datasets.

For each sequence *P* = *a*_1_ *a*_2_ ⋯ *a*_*L*_ ∈ *S*_*i*_, we select the most similar five sequences in each one of 6 sets {*p* | *p* ∈ *s*_*kc*_, *k* ≠ *i*},  *c* = 1,2, 3,4, 6,8 by PSI-Blastall. So we can get 30 features for each given sequence *P* based on the Evalue's index of the scientific notation from the results of the tool.

The sequence motifs can describe many properties of protein, such as transcription factor binding sites, splice junctions, and protein-protein interaction sites. Both GIBBS and GLAM2 are employed to find motifs from our datasets. In the same way, for each sequence *P* ∈ *S*_*i*_, we get the motifs of each one of 6 sets {*p* | *p* ∈ *s*_*kc*_,  *k* ≠ *i*},  *c* = 1,2, 3,4, 6,8 by both GLAM2 and GIBBS, denoted as follows, respectively:

(3) $$\begin{array}{c} M_{c}^{P_{\text{GLAM}2}},\;c=1,2,3,4,6,8,\\ M_{c}^{P_{\text{GIBBS}}},\;c=1,2,3,4,6,8. \end{array}$$

In fact, there are many gaps in some motifs generated by GLAM2 so that we need to preprocess these motifs as follows.

- If a motif has more than five consecutive gaps, we delete those gaps and divide this motif into two new motifs.
- If the AAs of a motif are less than five, we delete it.

Then we get updated

(4) $$\begin{array}{c} M_{c}^{P_{\text{GLAM}2}},\;c=1,2,3,4,6,8. \end{array}$$

We use the modified Smith-Waterman dynamic programming (SW-DP) algorithm to make sequence alignment between the given sequence *P* and each one of *M*_*c*_^*P*_GLAM2_^,  *c* = 1,2, 3,4, 6,8. The given sequence *P* acquires the five highest alignment scores from each of *M*_*c*_^*P*_GLAM2_^,  *c* = 1,2, 3,4, 6,8, so that we can get 30 more features for the given sequence. The specific procedure is as follows. In fact, each position of each motif generated by GLAM2 possibly has more than one AA after preprocessing. We use

(5) $$\begin{array}{c} M_{\text{GLAM}2}=m_{1}m_{2}\cdots m_{n} \end{array}$$

to represent a motif with *n* length, where *m*_*i*_ = {*b*_*ij*_} and *b*_*ij*_ may be one of 20 common AAs or a gap. For the protein sequence *P* = *a*_1_ *a*_2_ ⋯ *a*_*L*_, the penalty function is defined as

(6) $$\begin{array}{c} d_{\text{GLAM}2}\left(m_{i},a_{j}\right)=\{\begin{array}{cc} 1 & \text{if}\;\;a_{j}\in m_{i},\;\;a_{j}\neq \text{gap }\\ 0 & \text{if}\;\;a_{j}\in m_{i},\;\;a_{j}=\text{gap }\\ -1 & \text{if}\;\;a_{j}\notin m_{i},\;\;a_{j}\neq \text{gap }\\ -\frac{1}{3} & \text{if}\;\;a_{j}\notin m_{i},\;\;a_{j}=\mathrm{gap}. \end{array} \end{array}$$

Then we use the SW-DP algorithm to compute the alignment score between *P* and *M*_GLAM2_.

In addition, GIBBS can find a motif like

(7) $$\begin{array}{c} M_{\text{GIBBS}}=t_{1}t_{2}\cdots t_{n} \end{array}$$

for each one of *M*_*c*_^*P*_GIBBS_^,  *c* = 1,2, 3,4, 6,8, where

(8) $$\begin{array}{c} t_{i}={\left(p_{i}^{b_{1}},p_{i}^{b_{2}},\ldots ,p_{i}^{b_{21}}\right)}^{T},\;i=1,2,3,\ldots ,n \end{array}$$

represent probabilities of 20 common AAs and gap in the position *i*, and

(9) {bj, j=1,2,…,21}={A,R,N,D,C,Q,E,G,H,I,L,K,M,  F,P,S,T,W,Y,V,–}.

For the protein sequence *P* = *a*_1_ *a*_2_ ⋯ *a*_*L*_, the penalty function is defined as

(10) $$\begin{array}{c} d_{\text{GIBBS}}\left(t_{i},a_{j}\right)={p_{i}}^{a_{j}}. \end{array}$$

We employ the SW-DP algorithm to calculate the alignment score between *P* and *M*_GIBBS_ again, and then we gain other 6 features for the sequence *P* by GIBBS.

### 2.3. The Overall Design

Gaining a protein quaternary structure dataset, we design our method Quad-PRE from primary sequence as below.

1. Select the features based on properties of amino acid, PSSM, the secondary structure, the solvent accessible surface area, and the physicochemical property.
2. In addition, we divide our dataset equally into ten portions randomly, but making sure that every portion contains at least one element of each one of 6 states. And then we obtain the new features of each sequence using BLAST, GIBBS, and GLAM2, respectively.

**Figure 1:**
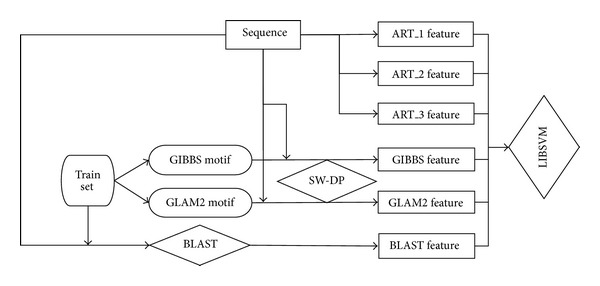
The diagram of Quad-PRE.

Our scheme is a hybrid method and we give a diagram for making it easy to follow, shown in Figure 1.

### 2.4. Classification

Support vector machine (SVM), which was shown to provide high quality predictions in classification, regression, and density estimation area, was implemented with LIBSVM [18] package. The support vector classification C-SVC is selected in this paper. There are several strategies to solve multiclass problem, such as one-versus-rest and one-versus-one. One-versus-rest strategy is used in this paper. The prediction performance was examined by *n*-fold cross validation, in which the training dataset is randomly divided into *n* subsets equally. The *n* − 1 subsets are used to train the model and the remaining one subset is used to evaluate the model, repeated *n* times. If *n* is the number of the samples, it was named jackknife test (or leave-one-out cross validation).

We designed a predictor with 10-fold cross validation. First of all, the input sequence is converted into the feature space, and then the corresponding features are passed to the classifier. The prediction class of the sequence that corresponds to one has the highest probability. Overall accuracy (ACC), the sensitivity or true positive rate (TPR), the false positive rate (FPR), the specificity (SPC), the precision (PPV), and Matthew's correlation coefficient (MCC) for each class are used to measure the prediction performance; they are defined as follows:

(11)ACC=(TP+TN)N,(12)TPR=TP(TP+FN),FPR=FP(FP+TN),SPC=TN(FP+TN)=1−FPR,PPV=TP(TP+FP),MCC=TP×TN−FP×FN(TP+FN)(TP+FP)(TN+FP)(TN+FN),

where TP is true positive number, TN is true negative, FP is false positive, FN is false negative, and *N* is total number of sequences. However, these metrics are not quite intuitive and easier-to-understand and we can adopt the formulation proposed recently to really understand them [19–21]. We also calculate the area under the ROC curve (AUC) to evaluate the predictions. Higher values of these measures indicate better quality of predictions.

## 3. Results and Discussion

### 3.1. Results and Comparison with Garian's QSE

The choice of the penalty factor *C* and the kernel function type is very important since SVM is sensitive to parameterization. In this paper, we consider the radial basis function (RBF) of kernel types following the Chang and lin [22]

(13) $$\begin{array}{c} K\left(x_{i},x_{j}\right)=\exp\left(-\gamma {||x_{i}-x_{j}||}^{2}\right), \end{array}$$

where *γ* is the width of the RBF function. To identify the optimal *C* and *γ*, a systematic grid search was conducted for

(14) C={0.1,0.2,0.5,1,2,5,10,20,50,100}γ={0.0025,0.005,0.01,0.02,0.04,0.08,0.16,0.32,0.64,  1.28,2.56,5.12,10.24}

by the 10-fold cross validation. Then we find the optimal *C* and *γ* are 0.1 and 0.01 with the average AUC value 0.704. With the best parameters, the average accuracy is 45.3% by 10-fold cross validation. The predicting matrix is as follows; the *rw*_*ij*_ is the number of the *i* class predicted as the *j* class

(15) $$\begin{array}{c} \text{RW}=\left(\begin{array}{cccccc} 285 & 55 & 12 & 1 & 5 & 8\\ 185 & 104 & 9 & 8 & 7 & 25\\ 24 & 9 & 13 & 0 & 1 & 6\\ 55 & 50 & 3 & 28 & 4 & 15\\ 18 & 20 & 6 & 0 & 16 & 7\\ 22 & 8 & 5 & 0 & 2 & 24 \end{array}\right). \end{array}$$

**Figure 2:**
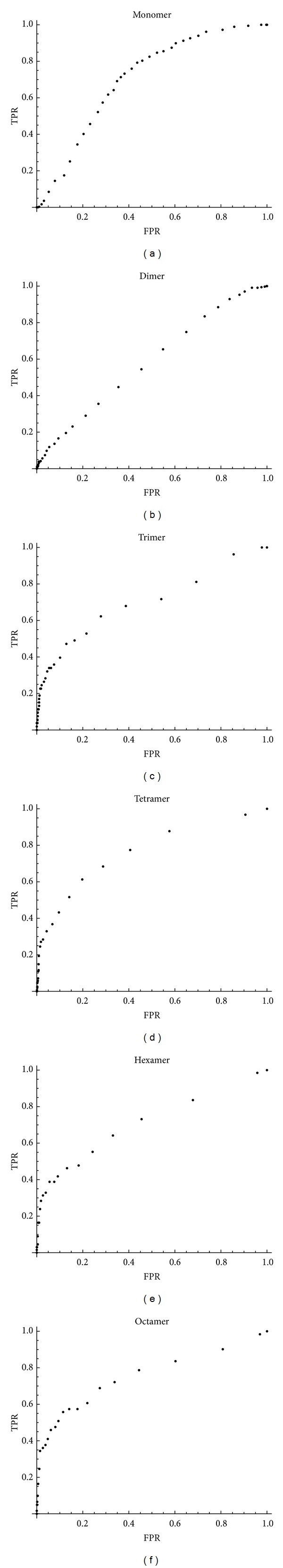
The ROC curves of six classes.

The TPR, SPC, PPV, MCC, and AUC of every class are shown in Table 3 and the ROC curves are shown in Figure 2. Following from Table 3, Quad-PRE achieved higher overall ACC 81.7%, especially higher accuracy 92.8%, 93.3%, and 90.6% on discrimination for trimer, hexamer, and octamer, respectively. And overall SPC is 87.0%, especially 96.5%, 99.0%, 98.0%, and 93.8% on discrimination for trimer, tetramer, hexamer, and octamer, respectively. These results show that our hybrid method has high accuracy and specificity.

**Figure 3:**
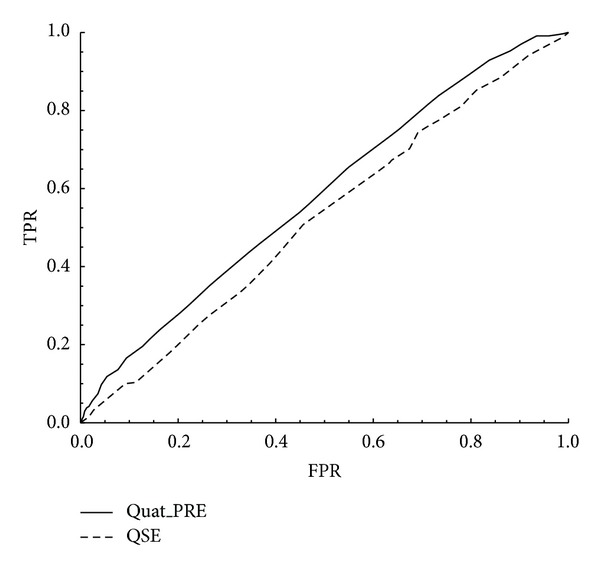
The ROC curves comparison Quad-PRE with Garian's QSE.

In addition, we can see that it is a little more difficult to predict dimer from Figure 2, because the AUC for predicting dimer is smaller than other oligomers. More specifically, the AUC of dimer is 0.582, while those of monomer, trimer, tetramer, hexamer, and octamer are 0.703, 0.702, 0.765, 0.711, and 0.758, respectively (see Table 2). However, when comparing with the predicted results of Garian's QSE [2] of classifying homodimer and nonhomodimer, the ACC, SPC, PPV, MCC, and AUC of Quad-PRE are all larger than QSE's, other than the TPR (see Table 4). Apparently, Quad-PRE performs better than QSE's (ROC curves of two methods are shown in Figure 3).

### 3.2. Discussion with Six Feature Groups

**Figure 4:**
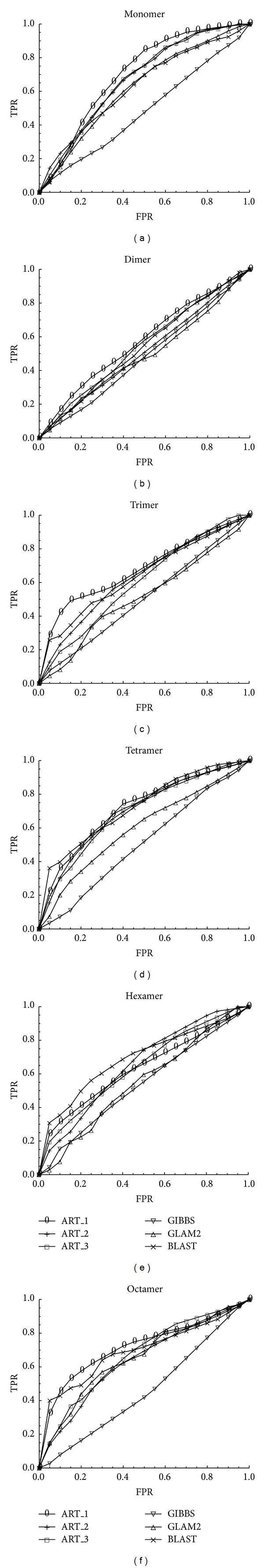
Comparison with the ROC curves of different classes for different feature groups.

For confirming our generated new features (TOTAL) can improve the prediction of protein quaternary structure attributes, we compared the results from TOTAL features with those from each one of the six feature sets (ART_1, ART_2, ART_3, BLAST, GLAM2, and GIBBS), which are shown in Table 5. The ROC curves for predicting every attribute by six sets are shown in Figure 4, respectively.

From Figure 4, we can see that the average AUC, ACC, TPR, SPC,and MCC of any of 6 features sets are all smaller than TOTAL features except the PPV. In particular, there are almost the same average SPC values for all feature sets. And the two feature sets from both GIBBS and GLAM2 all do not perform well in every metric. From Table 5 we also know that ART_1, BLAST, ART_1, ART_1, BLAST, and ART_1 play key roles in improving average ACC, TPR, SPC, PPV, MCC, and AUC of our method, respectively, because the corresponding values of them are close to those of TOTAL. These results mean each feature set contributes to the improvement of our hybrid method, especially ART_1 because the average ACC, TPR, SPC, PPV, MCC, and AUC from which are almost superior to others (see Table 5).

From the view of the average AUC, the importance of the six feature sets from high to low is ART_1, ART_2, ART_3, BLAST, GLAM2, and GIBBS (see Table 5). And the AUC values of ART_1, ART_2, and ART_3 for every protein attribute are almost larger than those of BLAST, GIBBS, and GLAM2 (see Figure 4). We think that the possible reason should be that the ART_1, ART_2, and ART_3 have much more features than BLAST, GIBBS, and GLAM2. And because similar sequences should have similar structures and functions, the features from BLAST are superior to those from both GIBBS and GLAM2 in the performance of SVM.

## 4. Conclusions

To predict protein quaternary structure attribute is indeed a challenging problem. This paper presents a novel approach, that is, Quad-PRE, to solve the problem. Quad-PRE starts to consider the features about motifs generated by some tools. From analysis results, we know the number of these features is too little to play important roles in improving the performance of our method, so that we will attempt to find motif features more important in the future work. In addition, Quad-PRE is a multistate method classifying monomer, trimer, tetramer, hexamer, and octamer very well, while other previous methods to predict the quaternary structure attributes are mostly for 2 states.

In fact, the hybrid method Quad-PRE is high accuracy and specificity on discrimination for trimer, tetramer, hexamer, and octamer, respectively. But we compare the Garian's QSE with our Quad-PRE using our dataset for confirming our method is effective. The results show that our hybrid method performs better than Garian's QSE in predicting the homodimmer or not from metrics ACC, SPC, PPV, MCC, and AUC. In addition, we analyze the importance of the six feature sets. The result clearly shows that each of six features sets contributes to the improvement in prediction, especially the ART_1 feature set. And three new feature sets gained by BLAST, GLAM2, and GIBBS are all effective, because these motif features describe the inherent properties of the sequence inherent and the motifs in protein sequences can help us to understand the structure and function of the molecules the sequences represent [23].

In this paper, we did not consider feature selection because we want to make full use of each feature as many as possible and analyze the importance of each one of six features sets. We believe that future improvements will be possible by designing better sequence representations rather than applying more complex classifiers.

Since user-friendly and publicly accessible web-servers [24] represent the future direction for developing practically more useful predictors, we shall make efforts in our future work to provide a web-server for the method presented in this paper.

## Supplementary Material

Table S1 is the detailed definitions of the protein features considered in our method. Table S2 is the property groups used to aggregate similar amino acids, and Table S3 is the isoelectric point value, Fauchere-Pliska hydrophobicity value, Eisenberg hydrophobicity value and hydropathy value of the standard amino acids. Table S2 and Table S3 are used for generating the protein features.

## Figures and Tables

**Table 1:** The numbers of monomer, dimmer, trimer, tetramer, hexamer, and octamer in our benchmark dataset.

Total	Monomer	Dimer	Trimer	Tetramer	Hexamer	Octamer
1040	366	338	53	155	67	61

**Table 2:** Summary of the considered features, where *y* denotes one of the three secondary structure states and *x* denotes one of the 20 common AAs.

Feature sets	Description
Sequence-based (79)	Sequence length (1) Composition vector (20) The number of AAs in the sequence belonging to {R group, Electronic group, Hydrophobicity group, Exchange group} (18) First and second order composition moment vector (40)

PSSM-based (203)	From the PSSM matrix

Secondary structure (217)	Based on the features utilized in the PSI-Pred method (90) Based on the predicted secondary structure which describes collocation of helical and strand segments (127)

Average RSA based (23)	Average RSA of the residues with AA type *x* (20) Average RSA of the residues with secondary structure type *y* (3)

Average isoelectric point (1)	*pI* = 1/*N*∑_*i*=1_^*N*^ *pI*_*i*_, the *pI*_*i*_ values in the paper [11]

Auto-correlation functions based on FH_*i*_, EH_*i*_, and Hp indices (25)	*A*_*n*_^*a*^ = 1/(*N* − *n*)∑_*i*=1_^*N*−*n*^ *a*_*i*_ *a*_*i*+*n*_, where *a* defines the corresponding physicochemical properties, such as two hydrophobicity indices (the Fauchere-Pliska's (FH) with *n* = 1,2,…, 10 and the Eisenberg's (EH) *n* = 1,2,…, 6), and hydropathy (HP) index with *n* = 1,2,…, 9.

Auto-correlation functions based on cumulative FH_*i*_ index (6)	*A*_*n*_^*a*^ = ∑_*i*=1_^*N*−*n*^(∑_*j*=1_^*i*^ *a*_*j*_) × (∑_*j*=1_^*i*+*n*^ *a*_*j*_)/(*N* − *n*), where *a* is the FH index with *n* = 1,2,…, 6.

Sum of hydrophobicities based on FH_*i*_ and EH_*i*_ (2)	*H*_sum_^*a*^ = ∑_*i*=1_^*N*^ *a*_*i*_, where *a* is the FH or the EH index.

R groups (5)	RG_*i*_, where *i* = 1 corresponds to nonpolar aliphatic AAs (AVLIMG), *i* = 2 to polar uncharged AAs (SPTCNQ), *i* = 3 to positively charged AAs (KHR), *i* = 4 to negative AAs (DE), and *i* = 5 to aromatic AAs (FYW); the composition percentage of each group in the sequence is computed

Electronic groups (5)	EG_*i*_, where *i* = 1corresponds to electron donor AAs (DEPA), *i* = 2 to weak electron donor AAs (LIV), *i* = 3 to electron acceptor AAs (KNR), *i* = 4 to weak electron acceptor AAs (FYMTQ), and *i* = 5 to neutral AAs (GHWS); the composition percentage of each group in the sequence is computed

Blast based (30)	Refer to subsection “*Features*”

GLAM2-based (30)	Refer to subsection “*Features*”

GIBBS-based (6)	Refer to subsection “*Features*”

**Table 3:** Predicted results with *C* = 0.1 and gamma = 0.01.

	Monomer	Dimer	Trimer	Tetramer	Hexamer	Octamer	Average
ACC	63.0%	63.8%	**92.8%**	87.0%	**93.3%**	**90.6%**	**81.7%**
TPR	77.9%	30.8%	24.5%	18.1%	23.9%	39.3%	35.7%
SPC	54.9%	79.8%	**96.5%**	**99.0%**	**98.0%**	**93.8%**	**87.0%**
PPV	48.4%	42.3%	27.1%	75.7%	45.8%	28.2%	44.6%
MCC	0.316	0.116	0.220	0.328	0.299	0.284	0.260
AUC	**0.703**	0.582	**0.702**	**0.765**	**0.711**	**0.758**	**0.704**

**Table 4:** Comparison with Garian's method.

	ACC	TPR	SPC	PPV	MCC	AUC
Quad-PRE	**63.8%**	30.8%	**79.8%**	**42.3%**	**0.116**	**0.582**
QSE	46.2%	73.8%	32.6%	34.7%	0.065	0.522

**Table 5:** Comparison with results are generated by different feature groups.

	ART_1	ART_2	ART_3	BLAST	GLAM2	GIBBS	Total
ave-ACC	**42.4%**	38.5%	39.9%	34.9%	23.7%	30.6%	**43.5%**
ave-TPR	23.9%	21.8%	23.2%	**33.0%**	22.7%	15.3%	**35.7%**
ave-SPC	**85.5%**	84.7%	85.1%	85.3%	84.5%	82.6%	**87.0%**
ave-PPV	**50.5%**	27.4%	28.9%	35.2%	20.5%	10.2%	**44.6%**
ave-MCC	0.153	0.090	0.111	**0.189**	0.051	−0.024	**0.260**
ave-AUC	**0.680**	0.662	0.661	0.660	0.573	0.510	**0.704**

## References

[1] Anfinsen CB, Haber E, Sela M, White FH (1961). The kinetics of formation of native ribonuclease during oxidation of the reduced polypeptide chain. *Proceedings of the National Academy of Sciences of the United States of America*.

[2] Garian R (2001). Prediction of quaternary structure from primary structure. *Bioinformatics*.

[3] Zhang S-W, Pan Q, Zhang H-C, Zhang Y-L, Wang H-Y (2003). Classification of protein quaternary structure with support vector machine. *Bioinformatics*.

[4] Chou K-C, Cai Y-D (2003). Predicting protein quaternary structure by pseudo amino acid composition. *Proteins: Structure, Function and Genetics*.

[5] Shi JY, Pan Q, Zhang SW (2006). Classification of protein homo-oligomers using amino acid composition distribution. *Acta Biophysica Sinica*.

[6] Carugo O (2007). A structural proteomics filter: prediction of the quaternary structural type of hetero-oligomeric proteins on the basis of their sequences. *Journal of Applied Crystallography*.

[7] Levy ED (2007). PiQSi: protein quaternary structure investigation. *Structure*.

[8] Xiao X, Lin W-Z (2009). Application of protein grey incidence degree measure to predict protein quaternary structural types. *Amino Acids*.

[9] Xiao X, Wang P, Chou K-C (2011). Quat-2L: a web-server for predicting protein quaternary structural attributes. *Molecular Diversity*.

[10] Sun X-Y, Shi S-P, Qiu J-D, Suo S-B, Huang S-Y, Liang R-P (2012). Identifying protein quaternary structural attributes by incorporating physicochemical properties into the general form of Chou’s PseAAC via discrete wavelet transform. *Molecular BioSystems*.

[11] Kurgan L, Chen K (2007). Prediction of protein structural class for the twilight zone sequences. *Biochemical and Biophysical Research Communications*.

[12] Mizianty MJ, Kurgan L (2009). Modular prediction of protein structural classes from sequences of twilight-zone identity with predicting sequences. *BMC Bioinformatics*.

[13] Zhang H, Zhang T, Gao J, Ruan J, Shen S, Kurgan L (2012). Determination of protein folding kinetic types using sequence and predicted secondary structure and solvent accessibility. *Amino acids*.

[14] Altschul SF, Madden TL, Schaffer AA, Zhang J, Zhang Z (1997). Gapped BLAST and PSI-BLAST: a new generation of protein database search programs. *Nucleic Acids Research*.

[15] Bryson K, McGuffin LJ, Marsden RL, Ward JJ, Sodhi JS, Jones DT (2005). Protein structure prediction servers at University College London. *Nucleic Acids Research*.

[16] Huang J-T, Cheng J-P, Chen H (2007). Secondary structure length as a determinant of folding rate of proteins with two- and three-state kinetics. *Proteins: Structure, Function and Genetics*.

[17] Ahmad S, Gromiha MM, Sarai A (2003). Real value prediction of solvent accessibility from amino acid sequence. *Proteins: Structure, Function and Genetics*.

[18] Hsu C-W, Lin C-J (2002). A comparison of methods for multiclass support vector machines. *IEEE Transactions on Neural Networks*.

[19] Chen W, Feng PM, Lin H, Chou K-C (2013). iRSpot-PseDNC: identify recombination spots with pseudo dinucleotide composition. *Nucleic Acids Research*.

[20] Qiu WR, Xiao X, Chou KC (2014). iRSpot-TNCPseAAC: identify recombination spots with trinucleotide composition and pseudo amino acid components. *International Journal of Molecular Sciences*.

[21] Xu Y, Ding J, Wu LY, Chou KC (2013). iSNO-PseAAC: predict cysteine S-nitrosylation sites in proteins by incorporating position specific amino acid propensity into pseudo amino acid composition. *PLoS ONE*.

[22] Chang C-C, Lin C-J (2011). LIBSVM: a library for support vector machines. *ACM Transactions on Intelligent Systems and Technology*.

[23] Bailey TL (2007). Discovering sequence motifs. *Methods in Molecular Biology*.

[24] Xiao X, Lin WZ, Chou KC (2013). Recent advances in predicting protein classification and their applications to drug development. *Current Topics in Medicinal Chemistry*.

