# Supplementary material for: Quad-PRE: A Hybrid Method to Predict Protein Quaternary Structure Attributes
Source: Comput Math Methods Med. 2014 May 18;2014:715494. doi: 10.1155/2014/715494 (PMC4052169; doi:10.1155/2014/715494)
Supplement: Supplementary file 1 — Table S1 is the detailed definitions of the protein features considered in our method. Table S2 is the property groups used to aggregate similar amino acids, and Table S3 is the isoelectric point value, Fauchere-Pliska hydrophobicity value, Eisenberg hydrophobicity value and hydropathy value of the standard amino acids. Table S2 and Table S3 are used for generating the protein features. [file 715494.f1.doc]

**Table S1.** The definition of the considered features

| **Feature sets** | **Description** |
| --- | --- |
| Sequence based (79) | ● sequence length:$N$. (1)  ● Composition vector:$CV_{i}=\frac{x_{i}}{N}$, where$x_{i}$represents the frequency of the$i$th AA in the sequence. (20)  ●$Comp\_GR_{jk}=\frac{count(AA\in GR_{jk})}{N}$, the number of AAs in the sequence belonging to $GR_{jk}$ where j ∈ {R group, Electronic group, Hydrophobicity group, Exchange group} and k is a particular subgroup (e.g., hydrophobic and hydrophilic), see **Table S2**, normalized by the sequence length. (18)  ●The first and second order composition moment vector: $CMV_{i}^{k}=\frac{\sum_{i,j=1}^{x_{i}} n_{ij}^{k}}{\prod_{d=1}^{k} (N-d)}$where $n_{ij}$ represents the$j$th position of the $i$th AA, and $k=1,2$ is the order of the CMV. (40) |
| PSSM based:Two position specific scoring matrices are generated by PSI BLAST. They are the conservation scores and probability of occurrence of a given AA at given position in the sequence, respectively denoted as $PSSMcons_{lm}$and$PSSMprob_{lm}$, where $l=1,2,...,SeqLen$represents the position in the sequence and $m=1,2,...,20$denotes one of the substitution positions that correspond to the twenty AAs . (203) | ●based on $PSSMcons_{lm}$(101)  $Seq\_CS\_\{AA_{i}\}=\frac{\sum_{l=1}^{N} PSSMcons_{li}}{N}$,where $AA_{i}$ represents the $i$th AA.(20) $Seq\_CS\_GR_{jk}=\frac{\sum_{l=1}^{N} \sum_{m\in GR_{jk}} PSSMcons_{lm}}{N}$ , sum of normalized $PSSMcons_{lm}$ values where l = 1, 2...N and $m=GR_{jk}$ (all AA types that belong to $GR_{jk}$, see **Table S2**) divided by the sequence length. (18 ) $Ach\_CS\_\{AA_{i}\}=\frac{\sum_{l=1}^{N} if AA(l)=AA_{i}, then PSSMcons_{li}, else 0}{N}$   ,where $AA(l)$ represents the $l$th position AA in the sequence.(20)  $Ach\_CS=\sum_{i=1}^{20} Ach\_CS\{AA_{i}\}$(1)  $Max\_CS\_\{AA_{i}\}=\frac{\sum_{l=1}^{N} if AA(l)=AA_{i} , then \max_{m=0}^{20}(PSSMcons_{lm}) , else 0}{N}$(20)  $Max\_CS=\sum_{i=1}^{20} Max\_CS\{AA_{i}\}$(1)  $Max\_Ach\_CS\_\{AA_{i}\}=\frac{\sum_{l=1}^{N} if AA(l)=AA_{i} then[\max_{m=0}^{20}(PSSMcons_{lm})-PSSMcons_{li}] else 0}{N}$(20)  $Max\_Ach\_CS=\sum_{i=1}^{20} Max\_Ach\_CS\{AA_{i}\}$(1)  ●based on $PSSMprob_{lm}$(102) $Seq\_\text{Prob}\_GR_{jk}=\frac{\sum_{l=1}^{N} \sum_{m\in GR_{jk}} PSSMprob_{lm}}{N}$ (18 )  $Ach\_\text{Prob}\_\{AA_{i}\}=\frac{\sum_{l=1}^{N} if AA(l)=AA_{i} then PSSMprob_{li} else 0}{N}$(20)  $Ach\_\text{Prob}=\sum_{i=1}^{20} Ach\_\text{Prob}\{AA_{i}\}$(1)  $Max\_\text{Prob}\_\{AA_{i}\}=\frac{\sum_{l=1}^{N} if AA(l)=AA_{i} , then \max_{m=0}^{20}(PSSMprob_{lm}) , else 0}{N}$(20)  $Max\_\text{Prob}=\sum_{i=1}^{20} Max\_\text{Prob}\{AA_{i}\}$(1)  $Max\_Ach\_\text{Prob}\_\{AA_{i}\}=\frac{\sum_{l=1}^{N} if AA(l)=AA_{i} then[\max_{m=0}^{20}(PSSMprob_{lm})-PSSMprob_{li}] else 0}{N}$(20)  $Max\_Ach\_\text{Prob}=\sum_{i=1}^{20} Max\_Ach\_\text{Prob}\{AA_{i}\}$(1)  (20)  $Ent=\sum_{i=1}^{20} Ent\_\{AA_{i}\}$(1) |
| Secondary structure:  The secondary structure is predicted as E(strand), H (helix) and C(coil) by PSI-Pred (217) | ●Based on the features utilized in the PSI-Pred method(90)  $Content\_\{SS_{i}\}=\frac{count(AA(l):AA(l) predicted as SS_{i})}{N}$, where$SS_{i}$ represents one of E, H and C. (3)  $NormSegCount\_\{SS_{i}\}=\frac{count(SEG(SS_{i}) )}{\sum_{SS\in\{H,E,C\}} count(SEG(SS))}$, where $SEG(SS)$denotes the [continuous](app:ds:continuous) segment whose Secondary structures are the same as$SS$ (3) $MaxSegCount\_\{SS_{i}\}=\max( length(SEG(SS_{i})))$ (3)  (3) $AvgSegLength\_\{SS_{i}\}=average(length(SEG(SS_{i})))$ (3) $NormAvgSegLength\_\{SS_{i}\}=\frac{average(length(SEG(SS_{i})))}{N}$ (3)  $SegCount\_S\{H,E\}\_L=\frac{count(SEG: length(SEG(S) )\geq L)}{\sum_{SS\in\{H,E\}} count(SEG(SS))}$, where $S\in\{H,E\}$, $L=2,3,\ldots20$ (38)  $SegCount\_C\_L=\frac{count(SEG: length(SEG(C) )\geq L)}{\sum_{SS\in\{H,E,C\}} count(SEG(SS))}$, where $L=2,3,\ldots20$ (19)  $SegCount\_S\{H,E\}\_P=\frac{count(SEG: length(SEG(S) )\geq P*N)}{\sum_{SS\in\{H,E\}} count(SEG(SS))}$, where$S\in\{H,E\}$, $P=2\%,4\%,6\%,8\%,10\%$. (10)  $SegCount\_C\_P=\frac{count(SEG: length(SEG(C) )\geq P*N)}{\sum_{SS\in\{H,E,C\}} count(SEG(SS))}$, where$P=2\%,4\%,6\%,8\%,10\%$. (5)  ●Based on the predicted secondary structure which describe collocation of helical and strand segments(127)  $HH=\frac{count(HH)}{\sum_{SS\in\{H,E,C\}} count(SEG:SEG(SS))}$, the number of helix-coil-helix motifs divided by the total number of the secondary structure segments in a protein. (1)  $EE=\frac{count(EE)}{\sum_{SS\in\{H,E,C\}} count(SEG:SEG(SS))}$, the number of strand-coil-strand motifs divided by the total number of the secondary structure segments in a protein. (1)  $HE=\frac{count(HE)+count(EH)}{\sum_{SS\in\{H,E,C\}} count(SEG:SEG(SS))}$, the number of strand-coil-helix or helix-coil-strand motifs by the total number of the secondary structure segments in a protein. (1)  $\{HH,HE,EE\}\_L=\frac{count(\{HH,HE/EH,EE\}:length(Coil)\geq L)}{\sum_{SS\in\{H,E,C\}} count(SEG:SEG(SS))}$, the number of helix-coil-helix, helix-coil-strand/strand-coil-helix or strand-coil-strand motifs which include at least $L=2,3,\ldots,20$residues in the middle coil, divided by the total number of the secondary structure segments in a protein.(57)  $\{HH,HE,EE\}\_P=\frac{count(\{HH,HE/EH,EE\}:length(Coil)\geq P*N)}{\sum_{SS\in\{H,E,C\}} count(SEG:SEG(SS))}$, the number of helix-coil-helix, helix-coil-strand/strand-coil-helix or strand-coil-strand motifs which include at least $P=2,4,\ldots,10\%$ of a sequence length residues in the middle coil, divided by the total number of the secondary structure segments in a protein.(15)  $MaxHCH=\max(HC..H: count(H))$, the maximal number of helices among all helix-coil-helix-coil…coil-helix motifs, i.e., the maximal number of helix segments separated only by coils. (1)  $MaxECE=\max(EC..E: count(E))$, the maximal number of strands among all strand-coil-strand-coil…coil-strand motifs, i.e., the maximal number of strand segments separated only by coils. (1)  $AvgHCH=\frac{average(HC..H:count(H))}{\sum_{SS\in\{H,E,C\}} count(SEG:SEG(SS))}$, the average number of helices in all helix-coil-helix-coil…coil-helix motifs, divided by the total number of the secondary structure segments in a protein. (1)  $AvgECE=\frac{average(EC..E:count(E))}{\sum_{SS\in\{H,E,C\}} count(SEG:SEG(SS))}$, the average number of strands in all strand-coil-strand-coil…coil-strand motifs, divided by the total number of the secondary structure segments in a protein. (1)  $HCH\_L=\frac{count(HC..H:count(H)\geq L)}{\sum_{SS\in\{H,E,C\}} count(SEG:SEG(SS))}$, the number of helix-coil-helix-coil…coil-helix motifs with more than $L=2,\ldots,20$ helices, divided by the total number of the secondary structure segments. (19)  $HCH\_P=\frac{count(HC..H:count(H)\geq P*N)}{\sum_{SS\in\{H,E,C\}} count(SEG:SEG(SS))}$, the number of helix-coil-helix-coil…coil-helix motifs with more than$P=2,4,\ldots,10\%$ of all helices in a protein, divided by the total number of the secondary structure segments. (5)  $ECE\_L=\frac{count(EC..E:count(E)\geq L)}{\sum_{SS\in\{H,E,C\}} count(SEG:SEG(SS))}$, the number of strand-coil-strand-coil…coil-strand motifs with more than$L=2,\ldots,20$strands, divided by the total number of the secondary structure segments. (19)  $ECE\_P=\frac{count(EC..E:count(E)\geq P*N)}{\sum_{SS\in\{H,E,C\}} count(SEG:SEG(SS))}$，the number of strand-coil-strand-coil…coil-strand motifs with more than$P=2,4,\ldots,10\%$ of all helices in a protein, divided by the total number of the secondary structure segments. (5) |
| Average RSA based (23) | Average RSA of the residues with AA type$x$(20)  Average RSA of the residues with secondary structure type$y$ (3) |
| Average isoelectric point (1) | $pI=\frac{1}{N}\sum_{i=1}^{N} pI_{i}$, the $pI_{i}$ values in **Table S3**. (1) |
| Auto-correlation functions based on $FH_{i}$,$EH_{i}$ and $Hp_{i}$ indices (25) | $A_{n}^{a}=\frac{1}{N-n}\sum_{i=1}^{N-n} a_{i}a_{i+n}$, where $a$ defines the corresponding physicochemical properties, such as two hydrophobicity indices (the Fauchere-Pliska’s (FH) with $n=1,2,\cdots,10$ and the Eisenberg’s (EH) $n=1,2,\cdots,6$), and hydropathy (HP) index with $n=1,2,\cdots,9$. The value$a_{i}$every AA of is shown in **Table S3**.(25) |
| Auto-correlation functions based on cumulative$FH_{i}$ index (6) | $A_{n}^{a}=\frac{\sum_{i=1}^{N-n} (\sum_{j=1}^{i} a_{j})\times(\sum_{j=1}^{i+n} a_{j})}{N-n}$, where$a$is the FH index with $n=1,2,\cdots,6$(6) |
| Sum of hydrophobicities based on $FH_{i}$ and $EH_{i}$ (2) | $H_{sum}^{a}=\sum_{i=1}^{N} a_{i}$, where $a$ is the FH or the EH index (2) |
| R groups (5) | RG, where$i=1$corresponds to non-polar aliphatic AAs (AVLIMG), $i=2$to polar uncharged AAs (SPTCNQ), $i=3$to positively charged AAs (KHR), $i=4$to negative AAs (DE), and $i=5$to aromatic AAs (FYW); the composition percentage of each group in the sequence is computed (5) |
| Electronic groups (5) | EG, where $i=1$corresponds to electron donor AAs (DEPA), $i=2$to weak electron donor AAs (LIV), $i=3$to electron acceptor AAs (KNR), $i=4$to weak electron acceptor AAs (FYMTQ), and $i=5$to neutral AAs (CGHWS); the composition percentage of each group in the sequence is computed (5) |
| Blast-based (30) | Refer to subsection “***Features***” in MS (30) |
| GLAM2-based (30) | Refer to subsection “***Features***” in MS (30) |
| GIBBS-based (6) | Refer to subsection “***Features***” in MS (6) |

**Table S2.** The property groups used to aggregate similar amino acids

| **R groups** | | **Electronic groups** | |
| --- | --- | --- | --- |
| Non-polar aliphatic | A, I, L, V | Donors | A, D, E, P |
| Glycine | G | Weak donors | I, L, V |
| Non-polar | F, M, P, W | Acceptors | K, N, R |
| Polar uncharged | C, N, Q, S, T, Y | Weak acceptors | F, M, Q, T, Y |
| Polar charged | D, E, H, K, R | Neutral | C, G, H, S, W |
| **Hydrophobicity groups** | | **Exchange groups** | |
| Hydrophobic | A, C, F, I, L, M, P, V, W, Y | Group 1 | H, R, K |
|  |  | Group 2 | D, E, N, Q |
|  |  | Group 3 | C |
| Hydrophilic | D, E, G, H, K, N, Q, R, S, T | Group 4 | S, T, P, A, G |
|  |  | Group 5 | M, I, L, V |
|  |  | Group 6 | F, Y, W |

**Table S3.** The isoelectric point(pI), Fauchere-Pliska hydrophobicity (FH)，Eisenberg hydrophobicity (EH) and hydropathy (Hp) of the standard amino acid

|  | **pI** | **FH** | **EH** | **Hp** |
| --- | --- | --- | --- | --- |
| Alanine | 6.01 | 0.42 | 0.62 | 1.8 |
| Cysteine | 5.07 | 1.34 | 0.29 | 2.5 |
| Aspartate | 2.77 | -1.05 | -0.9 | -3.5 |
| Glutamate | 3.22 | -0.87 | -0.74 | -3.5 |
| Phenylalanine | 5.48 | 2.44 | 1.19 | 2.8 |
| Glycine | 5.97 | 0 | 0.48 | -0.4 |
| Histidine | 7.59 | 0.18 | -0.4 | -3.2 |
| Isoleucine | 6.02 | 2.46 | 1.38 | 4.5 |
| Lysine | 9.74 | -1.35 | -1.5 | -3.9 |
| Leucine | 5.98 | 2.32 | 1.06 | 3.8 |
| Methionine | 5.47 | 1.68 | 0.64 | 1.9 |
| Asparagine | 5.41 | -0.82 | -0.78 | -3.5 |
| Proline | 6.48 | 0.98 | 0.12 | -1.6 |
| Glutamine | 5.65 | -0.3 | -0.85 | 3.5 |
| Arginine | 10.76 | -1.37 | -2.53 | -4.5 |
| Serine | 5.68 | -0.05 | -0.18 | -0.8 |
| Threonine | 5.87 | 0.35 | -0.05 | -0.7 |
| Valine | 5.97 | 1.66 | 1.08 | 4.2 |
| Tryptophan | 5.89 | 3.07 | 0.81 | -0.9 |
| Tyrosine | 5.67 | 1.31 | 0.26 | -1.3 |
